# Microsatellite Borders and Micro-sequence Conservation in ***Juglans***

**DOI:** 10.1038/s41598-019-39793-z

**Published:** 2019-03-06

**Authors:** Aziz Ebrahimi, Samarth Mathur, Shaneka S. Lawson, Nicholas R. LaBonte, Adam Lorch, Mark V. Coggeshall, Keith E. Woeste

**Affiliations:** 1 Department of Forestry and Natural Resources, Hardwood Tree Improvement and Regeneration Center, Purdue University, 715 State Street, West Lafayette, IN 47907 USA; 2 Department of Biological Sciences, Purdue University, West Lafayette, IN 47907 USA; 3 USDA Forest Service, Northern Research Station Hardwood Tree Improvement and Regeneration Center 715 West State St., West Lafayette, IN 47906 USA

## Abstract

Walnuts (*Juglans spp*.) are economically important nut and timber species with a worldwide distribution. Using the published Persian walnut genome as a reference for the assembly of short reads from six *Juglans* species and several interspecific hybrids, we identified simple sequence repeats in 12 *Juglans* nuclear and organellar genomes. The genome-wide distribution and polymorphisms of nuclear and organellar microsatellites (SSRs) for most *Juglans* genomes have not been previously studied. We compared the frequency of nuclear SSR motifs and their lengths across *Juglans*, and identified section-specific chloroplast SSR motifs. Primer pairs were designed for more than 60,000 SSR-containing sequences based on alignment against assembled scaffold sequences. Of the >60,000 loci, 39,000 were validated by e-PCR using unique primer pairs. We identified primers containing 100% sequence identity in multiple species. Across species, sequence identity in the SSR-flanking regions was generally low. Although SSRs are common and highly dispersed in the genome, their flanking sequences are conserved at about 90 to 95% identity within *Juglans* and within species. In a few rare cases, flanking sequences are identical across species of *Juglans*. This comprehensive report of nuclear and organellar SSRs in *Juglans* and the generation of validated SSR primers will be a useful resource for future genetic analyses, walnut breeding programs, high-level taxonomic evaluations, and genomic studies in *Juglandaceae*.

## Introduction

Walnuts (*Juglans*) are a genus of perennial trees and shrubs consisting of 21 species (Flora of North America) distributed in North America, South America, Eurasia, Central America, and the Caribbean^1^. Several members of the genus are important sources of edible nuts and wood, including *Juglans regia* (Persian walnut) which is grown as a crop in every country of the world with a temperate climate, and *Juglans nigra* (black walnut), the most valuable North American hardwood. Other North American *Juglans* species include *Juglans major* (Arizona black walnut), which grows in hot, arid areas near the border between the United States (U.S.) and Mexico, and *Juglans cinerea* (butternut), which along with black walnut grows in the eastern deciduous forests of the U.S. All New World *Juglans* belong to section Rhysocaryon. Aside from *J*. *regia* (sect. Dioscaryon), Asian *Juglans* belong to section Cardiocaryon. Species in this section include *Juglans mandshurica*, which is native to Korea and China, and *Juglans ailantifolia*, native to Japan. Species hybrids within *Juglans* are common, often fertile and vegetatively vigorous, and used as rootstocks for nut production or for timber.

Plant genomes contain a large proportion of non-coding repetitive DNA, including transposable elements, retroelements, non-LTR retroelements, tandem repeats, long and short interspersed nuclear elements, and micro- and mini-satellites^2,3^. Microsynteny among congeners for these types of repeated sequences is evidence for their conservation over evolutionary time and their role in genome evolution^2,3^. The characterization of synteny and microsynteny among congeners or other phylogenetically related groups for genes, or groups of genes, is an important objective of comparative genomics^4^. Flanking regions of microsatellites (SSRs) exemplify a type of synteny for non-genic and non-repetitive sequences that is important because when flanking regions are shared across species, PCR primers can be designed that amplify (presumably) homologous regions. Cross-species amplification of SSRs is used often in studies of species for which no SSRs have been published but SSRs from relatives are available^5,6^. The extent of genome-scale synteny and sequence conservation within genera at loci containing SSRs is not well understood^7^, although the sharing of LTR-retrotransposons and other repeat elements may be an important factor driving genome size variation across species and genera^8^. SSR markers are readily identified from both transcriptome and whole genome data^9^. Nuclear SSRs (nuSSRs) exhibit codominance, high multiplex potential, and many have high levels of polymorphisms. These characteristics make nuSSRs preferable for high throughput mapping, population genetic analysis, and marker-aided plant improvement techniques such as marker-assisted selection in tree breeding programs^10^, genetic diversity and gene flow analysis^11,12^, and quantitative trait loci (QTL) analysis^13^ in plant species.

The primary role of SSRs in plant evolution is unclear, but studies have shown that the distribution of motif frequencies and microsatellite density across the genome is not equal among species^14^ and seemingly non-random^15^. The dominant occurrence of motif patterns, motif repeats, and specific sequences and lengths in plant genomes likely result from selection pressures applied on that specific motif during evolution^16^. Aside from their use in population genetics and breeding (described above), SSRs can also be used for studies of synteny among species within one family and for comparisons of genome organization and evolutionary relationships across species. Except for *Juglans nigra* and *Juglans regia*^17–19^, few efforts have been made to generate SSR primer sets for other valuable *Juglans* species^20^.

Our study utilized paired-end sequencing (Illumina Inc., San Diego, CA) to identify and evaluate SSRs in 12 *Juglans* genomes. We sequenced and assembled genomes of several *Juglans* species to identify nuclear (nuSSR), mitochondrial (mtSSR), and chloroplast (cpSSR) microsatellites and compare their frequency and distribution within and across various *Juglans* genomes. We also aimed to evaluate SSR-flanking region sequence similarity across walnut species. Finally, we sought to design unique SSR primers for amplification in a single species or homologous loci in multiple *Juglans* species.

## Results and Discussion

### Library preparation and paired end sequencing data, frequency of motif types

**Figure 1:**
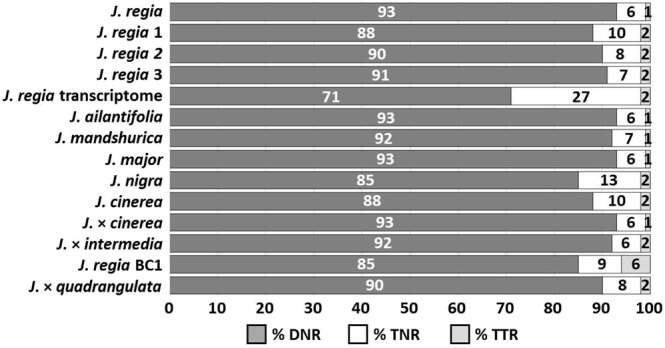
Frequency of SSR motifs in all examined genomes. (DNR, di-nucleotide; TNR, tri-nucleotide; TTR, tetra-nucleotide).

**Figure 2:**
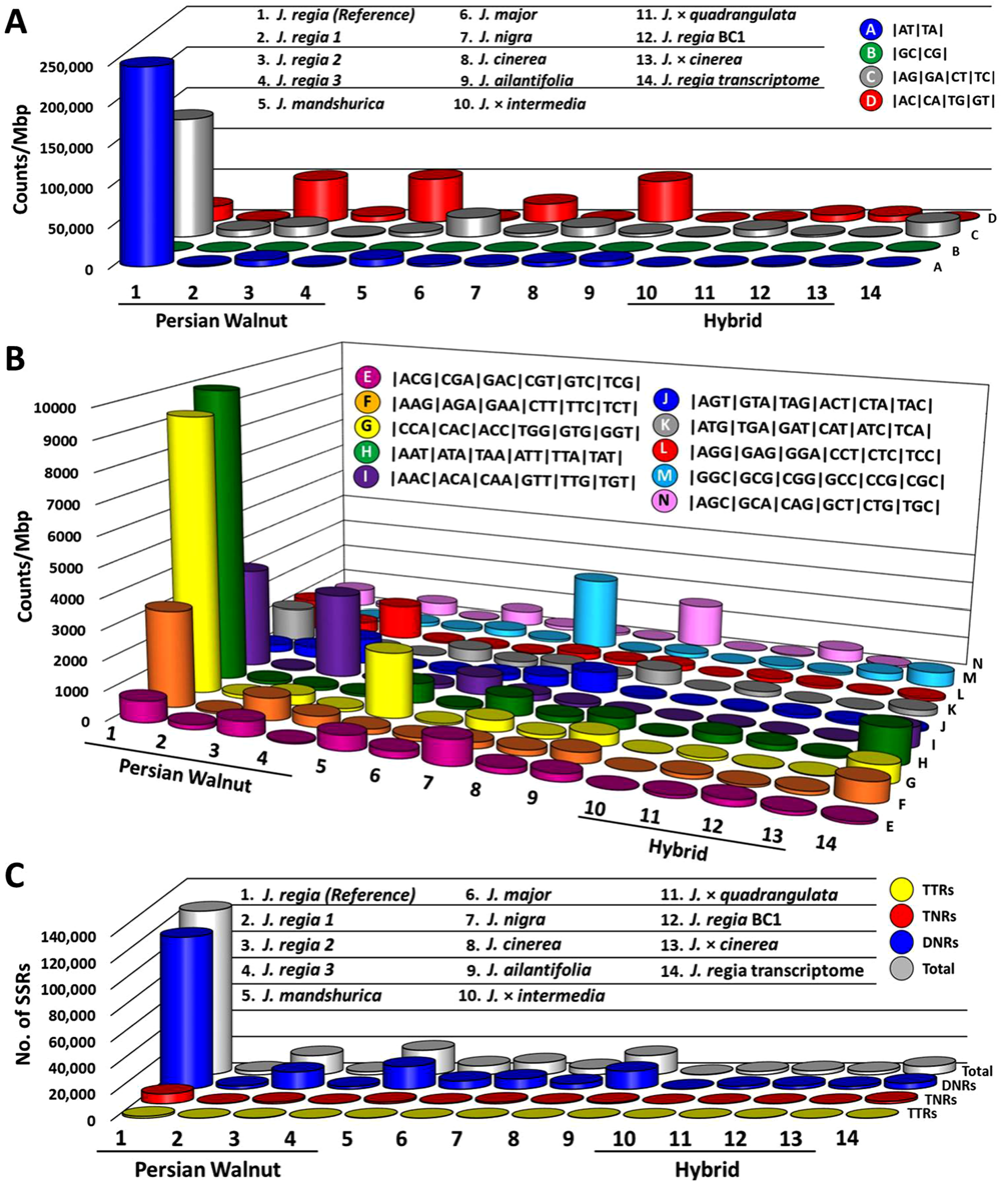
Motif type and frequency. (**A**) Abundance of di-nucleotide and (**B**) tri-nucleotide motifs. (**C**) Total numbers of nuSSRs within the nuclear genome of *Juglans spp*. and the Persian walnut (*J*. *regia*) transcriptome.

We used paired-end libraries to generate 977,379,442 reads (940 GB) of raw sequence data from all *Juglans* genotypes in our study. Quality control filtering yielded 787,083,476 reads (723 GB). Thus, 77% of data were used for downstream analyses (Table 1). The genomes of 12 walnut genotypes were sequenced and assembled using the draft nuclear genome, organellar genome, and transcriptome of *J*. *regia*^21^ as a reference to guide assemblies. Nuclear and organellar genomes of all 12 *Juglans* genotypes and the *J*. *regia* transcriptome were analyzed to identify simple sequence repeat (SSR) motifs. Motifs which repeated from two to four times and over a minimum length of 10 bp were selected for analysis (Supplementary Table S1). In *Juglans*, di-nucleotide and tri-nucleotide SSRs comprise 98% of total SSRs (Fig. 1). The AT/TA motif was the most frequent motif in the *J*. *regia* reference genome and across all of the 12 newly sequenced *Juglans* genomes (Fig. 2), but the frequency of this motif varied considerably; in *J*. *mandshurica*, *J*. *regia* and *J*. *major* it was 21%, 18%, and 4% respectively (Figs 1, 2, Supplemental Table S1). Our estimates of motif frequency were influenced by sequence quality and depth of coverage; for example, the frequency of a common motif was not consistent among different samples of *J*. *regia* or within sections of the genus. In general, the GC/CG motif was among the least represented of the dinucleotide repeats within *Juglans* genomes. The percentage of GC/CG motifs was relatively high in *J*. *nigra* (27%) from section *Rhysocaryon*, but much lower in another *Rhysocaryon*, *Juglans* major (10%). The frequency of GC/CG was surprisingly consistent in three *Cardiocaryon* species: *J*. *mandshurica* (17%), *J*. *cinerea* (17%), *J*. *ailantifolia* (17%), and among the samples of *J*. *regia* in section *Dioscaryon* (10%). Recent data indicated this specific repeat motif is rare in most hardwood trees, *Capsicum* species, *Arabidopsis*, rice, and wheat^17,22^. In *Capsicum*, the AG/CT repeat was the most abundant SSR^22^; this motif was also common in the *J*. *regia* reference, but not in the other *Juglans* genotypes (Fig. 2A), possibly because of low depth of coverage. The AAT/ATA motif was the most prominent tri-nucleotide motif found in *Juglans*. In monocots such as rice (*Oryza sativa*) and wheat (*Triticum spp*.), CCG/CGG comprised the greatest proportion of tri-nucleotide repeats^22^.

**Table 1:** Frequency of motifs and nuSSRs in all evaluated genomes.

Accession	Species	Total sequence^a^ (reads (million)	Coverage depth (X)	nuSSR ratio^b^	nuSSRs loci^c^	cpSSRs loci^d^	mtSSRs loci^d^
‘Chandler’ ^e^	*J*. *regia*	500	120	438,430	124,333	30	34
693	*J*. *regia-1*	82	12.63	13,236	2,808	30	34
694	*J*. *regia-2*	91	14.02	78,811	14,519	30	34
Lugar farm	*J*. *regia-3*	42	6.47	10,613	2,275	30	34
910	*J*. *mandshurica*	101	15.56	71,552	18,662	36	32
New Mexico	*J*. *major*	48	7.39	30,067	6,727	25	28
Purdue 1	*J*. *nigra*	131	20.18	34,927	9,029	12	26
OS-20	*J*. *cinerea*	73	11.25	20,650	4,587	16	32
1096	*J*. *ailantifolia*	61	9.40	64,193	14,597	24	36
654	*J*. × *intermedia*	5	1.0	1000	233	28	30
208	*J*. × *quadrangulata*	67	10.32	12,444	2,556	30	32
863	*J*. *regia* BC1	40	6.16	13,536	3,013	18	32
123 Rossville^f^	*J*. × *cinerea*	38	5.85	11,116	2,380	26	38
Transcriptome^g^	*J*. *regia*	0.7	—	23,596	6,379	—	—
Total		1,860		824,170	212,098	344	436

^a^Million reads; ^b^Ratio of cumulative sequence length of all SSR to genome size; ^c^Number of SSR present in nuclear genome (loci with designed primers); ^d^SSRs found in chloroplast and mitochondrial genomes; ^e^Published Persian walnut genome (*J*. *regia*); ^f^*J*. *cinerea* × *J*. *ailantifolia* backcross; ^g^*J. regia* transcriptome used for comparision.

Simple sequence repeat (SSR) motif frequency (among di-nucleotide, tri-nucleotide, and tetra-nucleotide motifs) decreased sharply with increased motif length in both the reference and the newly sequenced genomes (Fig. 1). For example, there were twice as many AT as AAT, and four times as many AG repeats as AAG (Supplementary Table S1). Di-nucleotide motifs account for 88.7% of nuSSRs while tri- and tetra-nucleotide motifs were significantly less abundant at 9.2% and 2%, respectively (Fig. 1). These data were similar to those from *Arabidopsis*, cucumber (*Cucumis sativus*), potato (*Solanum tuberosum*), tomato (*Solanum lycopersicum*)^22^, sorghum (*Sorghum bicolor*)^16^, and several hardwood tree genomes^17^. Simple sequence repeats (SSRs) with Tri-nucleotide motifs were the most common in monocot genomes^23^. Evaluation of nuclear genome motif frequency variation in other species showed that *Arabidopsis (Arabidopsis thaliana)*, cucumber (*Cucumis* s*ativa)*^22^, pine (*Pinus taeda* L.)^24^ and ten other hardwood tree species^17^ also showed a decline in motif numbers when motif lengths increased.

### nuSSR primers in *Juglans* genomes

A total of 824,170 loci containing SSR were identified in this study. We designed 205,000 SSR primers from the nuclear genome and 6,000 from the transcriptome (Table 1). Total SSR motif numbers from the reference genome and transcriptome were considerably higher than from other sequenced *Juglans* species (Fig. 2). For example, the number of SSRs in the *J*. *regia* reference genome was five times greater than that of *J*. *mandshurica* and seven times greater than *J*. *ailantifolia* (Table 1). The fewest SSRs were found in *J*. × *intermedia* (233) and *J*. × *cinerea* (2,380). Relatively few SSR loci were found in *J*. *major* and *J*. *nigra* (6,727 and 9,027) (Table 1*)*. *J*. *nigra* had three times more reads than *J*. *major*, but only about 1.5 X more SSRs. *J*. *quadrangulata* had more reads than *J*. *ailantifolia* but *J*. *ailantifolia* had 5x more SSRs. We identified a total of 189,208 di-nucleotide, 13,826 tri-nucleotide, and 1,990 tetra-nucleotide SSR loci among all 12 *Juglans* genomes examined (Supplementary Table S2a,b). The compiled *Juglans* SSR primer database resulting from this research will provide a rich resource for *Juglans* markers, enable development of in-depth linkage maps, and allow the fine-mapping of QTLs.

### Sequence similarities and synteny among SSR-flanking regions

**Figure 3:**
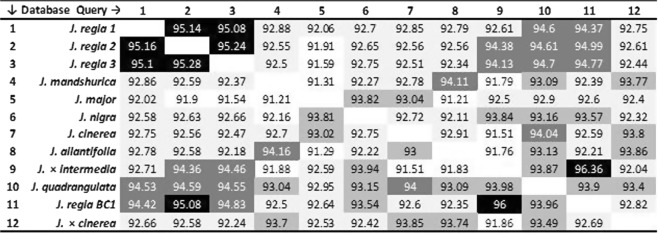
Predicted flanking regions of SSRs from all the walnut species were compared for pairwise mean sequence similarity and mean e-value using BLASTn.

We compared SSR flanking regions for sequence similarity and the presence of SNPs. BLASTN results indicated the pairwise mean sequence similarity of SSR flanking regions ranged from 91% to 96% across all *Juglans* genomes (Fig. 3). The species exhibiting the lowest average similarity to all other samples were *J*. *major* and *J*. *mandshurica* (91%, *e*-value = 1.006E-10) and the greatest pairwise similarity was between a *J*. *regia* backcross and *J*. × *intermedia* (96%). Similarity between pairs of *J*. *regia* samples was consistently about 95%. Similarity between all *J*. *regia* samples and related hybrids varied from 94% to 95%.

**Figure 4:**
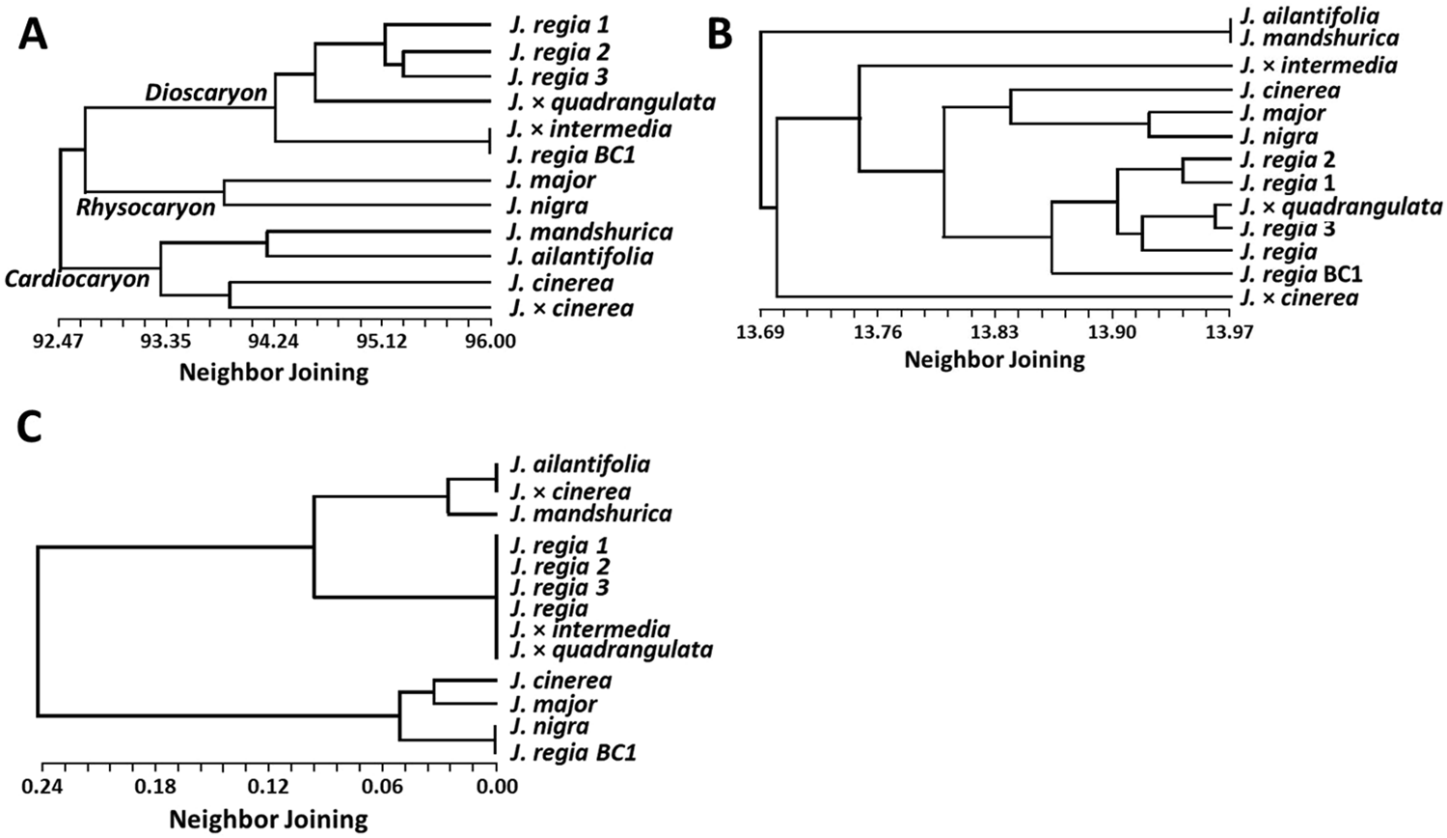
Phylogeny: (**A**) Phylogeny analyses based on mean similarity of all flanking region of nuSSRs obtained from BLASTN results. (**B**) Estimate of evolutionary divergence based on SSR-flanking region sequences. (**C**) Neighbor joining tree based on frequency of SSR motifs within the chloroplast.

We compared the similarity of each sample’s SSR-flanking region sequences based on BLASTN results. Similarity scores were used for neighbor joining analysis, and the resulting dendrograms showed *Juglans* species sorted into three main groups consistent with their conventional assignment to sections within the genus (Fig. 4A). A *Cardiocaryon* clade contained *J*. *ailantifolia* and *J*. *mandshurica*, *J*. *cinerea*, and *J*. × *cinerea*. *Juglans regia* genotypes and hybrids sorted into a *Dioscaryon* clade, while *J*. *nigra* and *J*. *major* sorted into a *Rhysocaryon* clade. Evolutionary analysis based on SSR-flanking region alignment (Fig. 4B) also sorted *Juglans* genotypes into their respective section with the exception that the closely-related *J*. *ailantifolia* and *J*. *mandshurica* could not be distinguished from one another^25^. The placement of *J*. × *cinerea*, *and J*. × *intermedia* based on a Maximum Composite Likelihood model (MCL), which estimates evolutionary divergence, was not the same as their placement based on BLASTN results (Fig. 4A,B). The sample identified as *J*. × *cinerea* was a hybrid of unknown but probably complex pedigree that included butternut and Japanese walnut. In our analysis, it sorted onto a branch between *J*. *ailantifolia* and species in other sections of the genus (Fig. 4B). Based on chloroplast data (Fig. 4C), *J*. × *cinerea* sorted closely with *J*. *ailantifolia*, probably because the hybrid contains a *J*. *ailantifolia* chloroplast. Our results are in the agreement with phylogeny results obtained using ITS (Internal Transcribed Spacer) and matK (megakaryocyte-associated tyrosine kinase)^26^. Our results showed that SSR flanking region sequences are a reliable method for phylogenetic evaluations of *Juglans* species and may represent an improvement over the marker systems used in previous studies because a large number of SSR flanking regions are available within genomes, are most often found in non-coding DNA (and so are likely to be neutral with respect to selection), and are scattered across the entire genome. About 16,000 flanking regions contributed to our phylogeny. Qi *et al*.^27^ reported that the majority of the genome (91%) is covered by neither tandem repeats nor indels, but much of the remaining genome (about 8%) is associated with SSR flanking regions. If these flanking regions are distributed across a random 8% of the genome, they may be useful targets for future analyses utilizing fosmid libraries or segmented genomes.

**Figure 5:**
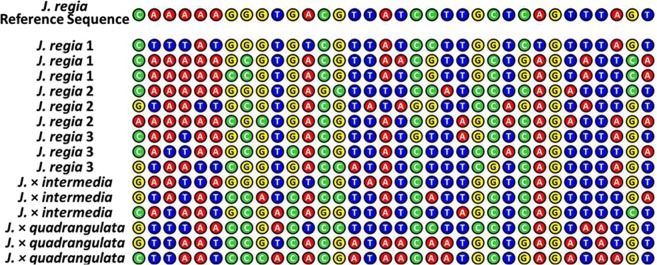
Genome-based SSR alignment. Typical alignment of SSR-flanking regions within *J*. *regia* and related hybrids).

**Figure 6:**
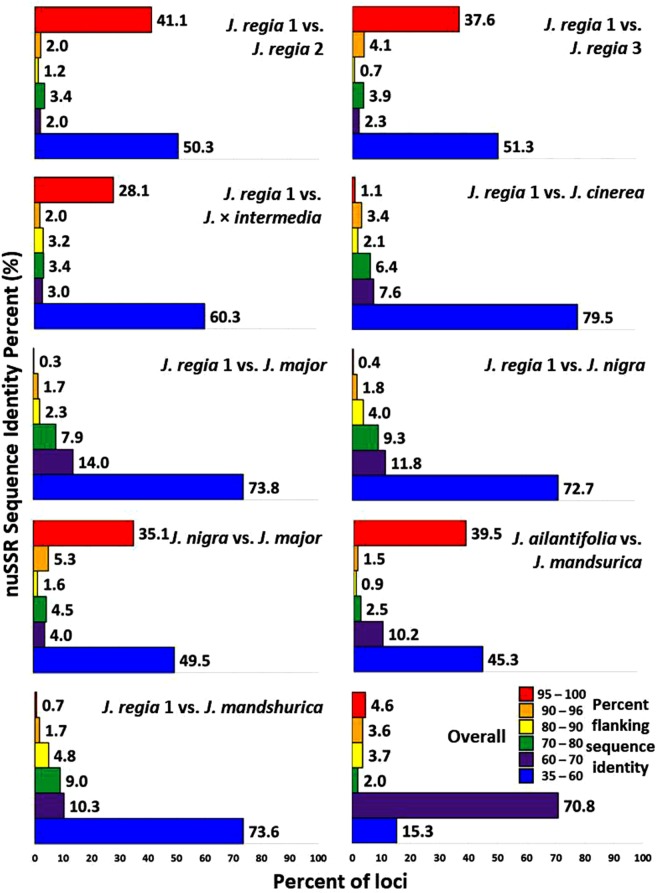
Percent flanking sequence identity. Percent of identical flanking-SSR sequences across *Juglans* genomes. Overall is the comparison of all genotypes versus all other genotypes.

Although our NGS data underwent adapter trimming before analysis, we cannot know how many SNPs in the flanking regions were true polymorphisms and how much of the sequence homology was homoplasious. Utilizing SNP calling with full coverage (100x) yields a 3% chance of having a read with SNP-like changes due to sequencing or assembly error^27^. In our data, the percentage of flanking regions that were identical to *J*. *regia* was low and varied among species 0.3 to 1.1%; thus, we were unable to design primers that were completely conserved across all species studied (Figs 5 and 6).

### Screening of SSR loci, development of unique shared nuSSR markers and genome duplication

To identify primer pairs that amplify only a single locus, primer sequences which occurred at multiple locations in the genome were filtered out as not useful for most applications^28^. We used a criterion of 100 percent identity in both primers for identifying duplicated loci, although it is likely that primers with less than 100% identity would cross-amplify non-target sites. Of the ~205,000 SSR-containing loci, we were able to design unique primers with >40% GC content within the flanking regions for ~60,000 loci. About 39,000 of these primer-containing loci were validated by e-PCR (Supplementary Table S4). The majority of validated single-locus primers identified in each species did not match a locus in any other *Juglans* species.

Of the 39,000 primer pairs, only 710 (1.8%) were 96–100% identical to a locus found in all *Juglans* (corresponding to a single nucleotide difference within a 20 bp primer), and no single primer sequence had a 100% match across all species studied (Fig. 6). In some cases, apparently identical loci in different species were similar enough in their flanking regions that we could design an upper primer with 100% identity and a lower primer with high homology (>95%). The use of wobble codes and adjustment of melting temperatures might make these primers practical as universal SSR primers for *Juglans*, or at least for all the species we evaluated. The greatest number of shared primer sets belonged to *J*. *ailantifolia* and *J*. *mandshurica*, with 37 shared primer pairs displaying 100% identity at a locus that was present only once in each species. Primers were designed for 63 regions conserved with 100% identity across at least two *Juglans* species (Supplementary Table S5). In all cases, complete primer sequence conservation was observed between only two species. Regions of shared homology with *J*. *regia*, the only species in the genus with a reference genome, could be useful in understanding genome evolution in *Juglans* and assist in comparative map development, especially if the SSRs associated with them are polymorphic in *J*. *regia*.

Based on pairwise comparisons among *J*. *regia* genomes, about half of the SSR regions in Persian walnut are evolving, and the other half remain highly conserved (Fig. 6). The proportion is the same, more or less, when comparisons are made within each section (for example, in *J*. *ailantifolia* versus *J*. *mandshurica* or *J*. *major vs*. *J*. *nigra*), but comparisons across sections (*J*. *regia* vs. *J*. *mandshurica* or *J*. *regia* vs. *J*. *major* or *J*. *cinerea* or *J*. *nigra*) showed that about 75% of the flanking regions have low similarity (30–60% identity). Across sections, very few loci (less than 1%) show high identity. This demonstrates why it is difficult to identify primers that will amplify well across the entire genus. Heterologous amplification has been demonstrated in *Juglans*, although the cross-species utility of primers varies by locus and by species^6^. The success of heterologous amplification using SSR primers depends generally upon the evolutionary distance between the original species and the tested species, with decreasing success as genetic distance increases^29^.

The fossil record places the radiation of the *Juglandaceae* to the Paleocene^30^ (approximately 56 to 66 MYA) and whole genome duplication may have occurred at this time, when the haploid number of chromosomes changed from 8 to 16. Within *Juglans*, it is unknown what percent of the genome is highly conserved across the genus. Factors leading to conservation, divergence or loss of flanking regions of SSRs in *Juglans* are probably similar to those in other species, but in general they are not well understood. The size of the genome, and number of retrotransposons and other mobile elements (which is not known for *Juglans*), may have an important role in genome duplication. Nevertheless, it seems clear that at least 710 (of 60,000) SSR loci in *Juglans* genomes are older than the most recent speciation in their lineage, as they were found across all *Juglan*s. Interestingly, about 708 loci were duplicated in *J*. *regia*, i.e., for these 708 SSRs there was 100% homology in the flanking region at two loci in the *J*. *regia* genome (Supplementary Table S8). These 708 loci may be so ancient as to predate the genome duplication event for *Juglans*. It is also possible their sequence identity is homoplasious. In some cases, the number of identical flanking regions was as great as 19, but duplicated or multiple copies of a flanking region was generally rare. The frequency of multiple copies of a flanking sequence in a genome was greater in *J*. *nigra* than in *J*. *regia* or *J*. *cinerea*, and considerably greater in *J*. *mandshurica* and *J*. *ailantifolia* than in other species we analyzed (Supplementary Table S8). A low percent of the genome was a shared flanking region with 100-percentage identity across 12 *Juglans* genomes; perfect sequence identity was found between two species only. Shared loci between two species most likely originated with the common ancestor of the two species in which they were found, and unique SSRs must have arisen later in evolutionary time. The relationship between SSR duplication within a genome and conservation of SSRs across genomes within a genus is not understood but could ultimately shed light on genome evolution. We suggest that loci with sufficient identity to share primers across two *Juglans* species probably arose prior to speciation, about 1 to 3 million years ago.

### Motif frequency in cpSSRs and mtSSRs

The extent of within species variation in chloroplasts and mitochondria in *Juglans* is poorly described, although there is some data based on reasonable sample sizes and sample distribution^31^. The reference genome and twelve additional walnut genotypes sequenced in this study revealed 230 chloroplast and 330 mitochondrial SSRs in the walnut genomes. Total density of SSRs in the chloroplast genome was significantly lower than mitochondrial genomes (*p* = *0*.*001*). There are no cpSSRs in *Juglans* chloroplasts known to be polymorphic within a species, emphasizing the low level of genetic variation found within *Juglans* chloroplasts. All chloroplast polymorphisms identified within *Juglans* species so far are indels or SNPs, many of which result in restriction site differences^32,33^. Many of these polymorphisms are in SSR-rich regions, but the documented polymorphism is not within the SSR. Comparison of all *Juglans* genomes showed both cpSSRs and mtSSRs exhibited very slow rates of evolution, which means that in *Juglans*, cpSSRs are good tools for high-level taxonomic evaluations^34–38^.

Di-, tri-, and tetra-nucleotide motifs were detected in both chloroplast and mitochondrial genomes, although their presence and frequency varied considerably among species. Motifs longer than three nucleotides in organellar genomes were generally rare and were termed “complex”. There were between three and six loci per chloroplast with complex nucleotide repeats, depending on walnut species (Supplemental Table S1). Complex motifs present in chloroplasts were unique for each species (Fig. 4C, Supplemental Table S1). The total numbers of motifs identified in chloroplast and mitochondrial genomes were small compared with nuSSRs but differences in the numbers of motifs among species and differences in the presence or absence of motifs among species may be great enough that they could be used for understanding plastid evolution (Supplementary Tables S1, S6, S7). The cpSSRs studied were rich in AT motifs. Among dinucleotide SSRs, AT/TA repeats were the most common (46%). Trinucleotide SSRs (ATT/ATA) were also present, but they were rare (1 to 3 motifs per genome). Trinucleotide variations appeared to be species dependent, with 1 to 2 motifs identified per species. *J*. *regia* and *J*. *mandshurica* displayed two ATT repeats and one ATA repeat, but *J*. *nigra*, *J*. *major*, and *J*. *cinerea* had only the two ATT repeats. *J*. *ailantifolia* and *J*. × *cinerea* (which likely contains a *J*. *ailantifolia* chloroplast) had only one ATT repeat.

All *J*. *regia* and *J*. *regia* hybrids (*J*. × *intermedia and J*. × *quadrangulata*) had similar repeat motifs in their chloroplast, and for this reason they formed a distinct clade **(**Fig. 4C**)**. For example, the ATAAA/TTTATA motif was only found in *J*. *regia* and hybrids of *J*. *regia* (*J*. × *intermedia* and in *J*. × *quadrangulata*), as expected, assuming the female parent of the hybrid was *J*. *regia*. The GATAA motif was found in *J*. *ailantifolia*, *J*. *mandshurica*, and *J*. × *cinerea* only, but the AATA motif was found in these species and *J*. *cinerea* (Fig. 4C, Supplemental Table S1). *J*. × *cinerea* was not joined with *J*. *cinerea*, which means the *J*. × *cinerea* sample contained a *J*. *ailantifolia* chloroplast, which is commonly observed (Fig. 4C). Chloroplast motifs were similar in *J*. *major*, *J*. *nigra*, and the *J*. *regia* backcross, with the exception that the *J*. *major* chloroplast did not contain a TAAA motif. Differences in read depth may have resulted in the apparent absence of motifs. The *J*. *regia* backcross was joined with *J*. *nigra* because *J*. *nigra* was used as a female in the first cross (Supplemental Table S1). Yi-heng *et al*.^39^ reported ATAAA motifs in *J*. *regia* and *J*. *sigillata* and AAGAT repeat motifs in *J*. *cathayensis*, *J*. *hopeiensis* and *J*. *mandshurica*. Whether these motifs are found in all lineages of these species is not yet established.

Chloroplast and mitochondrial SSRs were monomorphic, in sharp contrast with the nuclear genomes (Supplemental Table S1). In all 12 mitochondrial genomes studied, tri-nucleotide repeats were the most prevalent (42%), followed by tetra-nucleotide (35%) and di-nucleotide (23%) motifs. Complex motifs were absent from most species’ mitochondria, but complex motifs were present in the mitochondria of a few species (e.g., AGCA and TATC) (Supplemental Table S1). Most mitochondrial genome SSR motifs were conserved across all studied genomes; however GAA repeats were not found in *J*. *major*, *J*. *cinerea* or *J*. *nigra*. The remaining walnut species contained GAA motifs in addition to TCTT. The TAAA motif was found in *J*. *ailantifolia*, *J*. *cinerea*, *J*. × *cinerea and J*. *mandshurica* only. Although no complex motif was common to all the mitochondrial genomes we studied, there were several mtSSR motifs that were absent from some species and present in all the others.

### Data Archiving

All primer sequences were included as supplementary files.

## Conclusion

We developed databases containing lists of nuclear, chloroplast and mitochondrial loci containing SSRs from a total of 12 genotypes of six *Juglans* species. The depth and density of our marker database will assist researchers seeking to fill gaps in linkage-based genetic maps and improve the resolution of plant breeding approaches. Pairwise similarity of SSR flanking regions reflected known phylogeny. The further development of flanking sequences as sequence tagged sites would increase their utility. In particular, the maternally inherited cpSSRs showed high levels of sequence conservation and are useful for high-level taxonomic evaluations, including analyses of geographical origin, identification of distinct genetic lineages, and studies of dispersal. Mitochondrial motif patterns exhibited a lack of sequence diversity but showed variability in the number of repeats across species.

## Material and Methods

A flow chart of the methods process is available in the Supplementary files (Supplementary Fig. S1).

### Sample collection and DNA extraction

Tissue samples of 12 *Juglans* genotypes representing six species and four species hybrids were provided by the Hardwood Tree Improvement and Regeneration Center at Purdue University (HTIRC; www.htirc.org) (Table 1). Genomic DNA was extracted using a CTAB-based extraction method^40^.

### Library preparation, genomic sequencing, and sequence assembly

We constructed DNA sequencing libraries for each of the 12 walnut taxa and sequenced them using a single lane of paired-end reads at the Purdue Genomics Core (https://www.purdue.edu/hla/sites/genomics/) using the Illumina HighSeq2500 (Illumina Inc., San Diego, CA). Raw sequence reads were trimmed using Trimmomatic^41^. Contiguous sequence fragments were assembled *de novo* for each species with SOAP-denovo^42^ using trimmed and filtered reads. Whole genome, transcriptome, and organellar sequences from the Persian walnut genome were downloaded from the Walnut Genome Database (http://dendrome.ucdavis.edu/ftp/Genome_Data/genome/Reju/)^21^. Chloroplast genomes were constructed by assembling short reads to the Persian walnut chloroplast reference sequence^21^ using BWA^43^. Duplicates were flagged and sorted using the Picard tools software^44^ and SNPs were called using the Haplotype Caller tool from the Genome Analysis Toolkit (GATK)^45^. A similar approach was used to assemble the mitochondrial genomes.

### SSR primer pipeline

We used a modified Perl script from Staton *et al*.^17^ to identify microsatellites in our *Juglans* genomes. Only SSR loci containing perfect repeat units of 2–4 nucleotides were utilized for analysis. We set a minimum-length criterion for SSR analysis; 8–40 repeats for di-nucleotide SSRs, 7–30 repeats for tri-nucleotide SSRs, and 6–20 repeats for tetra-nucleotide SSRs. Our nuSSR primers met the following parameters: a product length of 100–200 bp, a primer size from 18 to 25 bp, annealing temperatures between 55–60 °C, and 40–60% GC content. Simple sequence repeats (SSRs) flanking regions were masked to exclude low complexity regions using Dustmasker^46^, and primers were designed using Primer3 (v2.3.5)^47^. Designed SSR primers were denoted ‘nuclear SSRs’ (nuSSRs) to distinguish them from motif sequences. Organellar and transcriptomic genome analyses utilized the bioinformatics pipeline described in Staton *et al*.^17^. Organellar SSR primers were designed using Primer 3 in batch mode^48^. Basic patterns (di-, tri-, and tetra-nucleotide) were identified in all studied genomes. Complex patterns were species-specific, and matched those previously reported for the chloroplast genome^39^. Unique motifs within the chloroplast genome, and the frequency of di-, tri-, and tetra- nucleotide repeats were counted based on the number of each motif in each species (Supplementary Table S1). Motif frequency values for each genotype were used to perform statistical and pairwise distance analyses of the genotypes using Ward’s method^49^. Phylogenetic analyses based on motif frequency for chloroplast genome were computed using Ntsys^50^.

### Similarity of SSR flanking regions among *Juglans* genomes

We performed one-on-one comparisons of all SSR-flanking region sequences identified using BLASTN (BLAST Command Line Applications User Manual 2016). Simple sequence repeat (SSRs) flanking sequences in all species were compared pairwise for sequence similarity and pairwise percent similarity for all comparisons were stored in a database. We then calculated the mean sequence similarity and mean *e*-value for all BLAST hit results. Mean sequence similarity between SSRs of different species was an indication of how concordant genetic markers and primer sequences of different walnut species were to each other. Pairwise similarity between species for all loci was used for neighbor joining. Mean *e*-values represent the probability of finding the same sequence in the database by chance, and are directly related to query sequence size. Shorter sequences have higher probabilities of occurring randomly based on mean sequence similarities (Fig. 3). Phylogenetic analysis was performed based on sequence similarity of SSR-flanking regions using Ntsys software^50^. To find SNP variation (Figs 5 and 6) and the level of heterozygosity across *Juglans* genomes, SSR flanking regions were aligned with MEGA6^51^. Mean distance within and between groups, genome heterozygosity and SNP variation were calculated with MEGA6.

To estimate the evolutionary divergence between sequences based on SSRs flanking regions, the number of base substitutions per site between sequences was measured. Analyses were conducted using the Maximum Composite Likelihood model (MCL). All positions containing gaps and missing data were eliminated. Evolutionary analyses were conducted in MEGA6^51^, which generated a matrix of mean distances among genomes which was analyzed using neighbor joining to represent the phylogeny. Phylogenetic analysis based on frequency of SSR motifs within the chloroplast was performed based on Neighbor joining methods and a tree was drawn using Ntsys software^50^.

### Filtering of unique SSRs and electronic polymerase chain reaction (e-PCR) validation

Primer sets were designed for all SSRs identified within the 12 *Juglans* genomes. To avoid untargeted amplification, primer sequences were matched against respective assembled scaffold sequences using BLASTN, and only the primer sets exactly matching a unique genomic region were retained. Primer sequences (forward or reverse) that aligned to multiple regions were filtered out^28^. The filtered primer pairs were designated ‘unique SSRs’ and were used for further downstream analysis. All remaining primer pair sets were sorted by their repeat number (>10 repeats) and GC content (40–60%). These unique primer sets were then validated by e-PCR^52^. Although we focused on primers that amplified a single locus, we also evaluated the frequency with which some primer sets could amplify multiple loci with the goal of understanding genome organization.

### Filtering shared SSRs among *Juglans* species

Primer pairs that were shared between two or more species were identified using custom Perl scripts and ClustalW (hybrids were not included in this analysis)^53^. All previously filtered forward and reverse primer sequences from each species were pairwise matched (>35% sequence identity). Retained primer sets showed 100% sequence identity with primer sets from a different species and displayed the same SSR-flanking region within both species. These SSR regions were considered ‘conserved’ between species.

## Supplementary information

Supplementary Fig. S1

Dataset1

Dataset 2a

Dataset 2b

Dataset 3

Dataset 4

Dataset 5

Dataset 6

Dataset 7

Dataset 8
