# Supplementary material for: Microsatellite Borders and Micro-sequence Conservation in Juglans
Source: Sci Rep. 2019 Mar 6;9:3748. doi: 10.1038/s41598-019-39793-z (PMC6403238; doi:10.1038/s41598-019-39793-z)

Title: **Microsatellite Borders and Micro-sequence Conservation in *Juglans***

Authors: **Aziz Ebrahimi, Samarth Mathur, Shaneka S. Lawson, Nicholas R. LaBonte, Adam Lorch, Mark V. Coggeshall, Keith E. Woeste.**

Supplementary Figure. S1

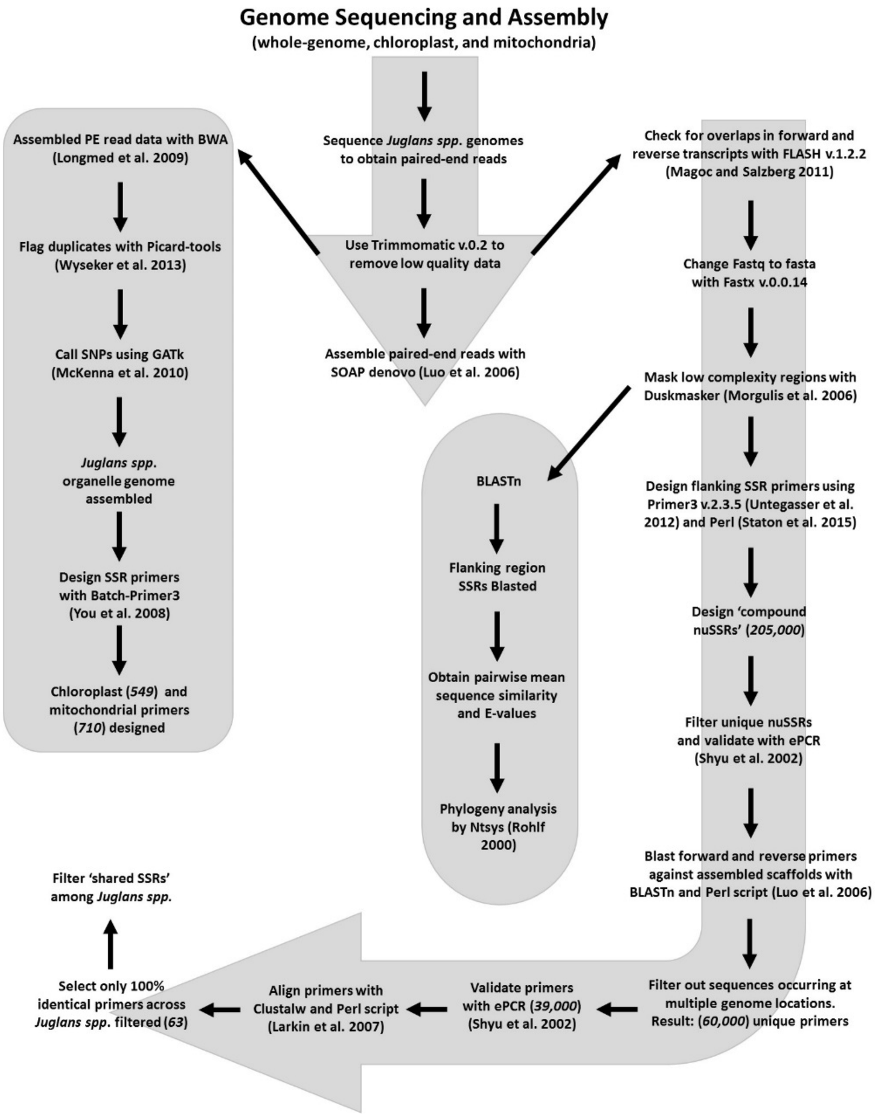

Supplement: Supplementary file 1 — Supplementary Fig. S1 [file 41598_2019_39793_MOESM1_ESM.pdf]
